# What Intensive care registries can teach us about outcomes

**DOI:** 10.1097/MCC.0000000000000865

**Published:** 2021-10-01

**Authors:** Abi Beane, Jorge I.F. Salluh, Rashan Haniffa

**Affiliations:** 1 Mahidol Oxford Tropical Medicine Research Unit, Oxford University, UK; 2 D’Or Institute for Research and Education (IDOR), Rio de Janeiro, Brazil; 3 Postgraduate program, Internal Medicine, Federal University of Rio de Janeiro, Rio de Janeiro, Brazil

**Keywords:** Critical care, intensive care, low-income and middle-income countries, outcomes, registries, benchmarking, patient centred outcomes, quality

## Abstract

**Purpose of review:**

Critical care registries are synonymous with measurement of outcomes following critical illness. Their ability to provide longitudinal data to enable benchmarking of outcomes for comparison within units over time, and between units, both regionally and nationally is a key part of the evaluation of quality of care and ICU performance as well as a better understanding of case-mix. This review aims to summarise literature on outcome measures currently being reported in registries internationally, describe the current strengths and challenges with interpreting existing outcomes and highlight areas where registries may help improve implementation and interpretation of both existing and new outcome measures.

**Recent findings:**

Outcomes being widely reported through ICU registries include measures of survival, events of interest, patient reported outcomes and measures of resource utilisation (including cost). Despite its increasing adoption, challenges with quality of reporting of outcomes measures remain. Measures of short-term survival are feasible, but those requiring longer follow-ups are increasingly difficult to interpret given the evolving nature of critical care in the context of acute and chronic disease management. Furthermore, heterogeneity in patient populations and in healthcare organisations in different settings makes use of outcome measures for international benchmarking at best complex, requiring substantial advances in their definitions and implementation to support those seeking to improve patient care.

**Summary:**

Digital registries could help overcome some of the current challenges with implementing and interpreting ICU outcome data through standardisation of reporting and harmonisation of data. In addition, ICU registries could be instrumental in enabling data for feedback as part of improvement in both patient centred outcomes and in service outcomes; notably resource utilisation and efficiency.

## Introduction

Critical care registries are synonymous with measurement of intensive care unit outcomes regionally and nationally [1]. Critical care registries are increasingly common globally. Recent high profile publications have highlighted the value and impact of registries in enabling continuous and replicable measure of quality and in supporting efforts to improve the effectiveness of healthcare internationally [2]. More recently registries are playing a pivotal role in capturing and reporting outcomes data in response to the global COVID-19 pandemic [3].

Historically the outcomes captured by registries (and registry based studies) were selected primarily based on the need for a basic evaluation of ICU performance and on research questions of interest, with some consideration given to the feasibility of capturing the desired outcomes within the study scope and available budget. In ICU, measures of mortality and morbidity dominated. The role of ICUs and their patient populations is however evolving. Admission for optimisation of physiology prior to complex surgery and for acute symptom management in the context of chronic disease and palliative care is now commonplace in many ICUs. Also, as governmental agencies, accreditation services and medical societies established quality of care and outcome measures, several ICU registries have incorporated those in their core data set. In addition there is growing recognition of the need to identify and define outcomes that consider perspectives beyond those of ICU clinicians and policy makers to include patients, their families and the wider multidisciplinary stakeholder group.[4,5]. This review aims to summarise literature on outcome measures currently being reported in registries internationally, describe current challenges with interpreting existing outcomes measured and highlight areas where registries may help improve implementation and interpretation of both existing and new outcome measures.

## What outcomes are currently being measured

Outcome measures of interest reported through registries can be considered under four broad categories: survival (including short-term and up to one year post discharge), events of interest, patient-reported outcomes (including longer term morbidity) and resource utilisation (including cost).[6].

### Survival measures

Survival measures remain the most frequently described and, perhaps, the most important measured outcome for many critical care registries. Survival measures, most notably all-cause ICU mortality, are defined and captured with relative consistency across national and international critical care registry groups. [3,7]. ICU and hospital mortality are frequently captured, although some registries also include some “fixed-time” (30, 60-day) reports on vital status. Whilst all-cause mortality is broadly relevant for most specialities and is useful for determining that a patient has died (as opposed to for example being lost to follow up), its utility in determining quality of care is limited. [2]. The latter requires the distinction between expected and excess mortality, and by nature of the distinction, identification of deaths which may have been avoided. However, reporting of procedure-related deaths including those related to a complication of the procedure or treatment for a complication remain widely absent from the majority of registries. [7].

Using risk-adjusted mortality represents a significant improvement to ensure the use of “mortality” as a quality of care indicator. The measurement of standardised mortality rates which are derived using prognostic scores is less consistent.[8,9,10]. Although all registries use standardised mortality rates (SMRs), internationally they vary in both the models used and the time points at which they are applied. The most frequently used are those based on APACHE (II,III and IV) and SAPS (2 and 3) which are used by registries in Europe, South America, Japan, whilst well established registries such as ICNARC (UK) and the Australia and New Zealand registry (ANZICS) use their own scoring systems- developed and validated specifically for their populations (ANZROD and ICNARC mortality model, respectively) [7,11,12]. More recently in emerging LMIC ICU registries including those in Asia (India, Nepal, Sri Lanka and Pakistan) and Latin America (Brazil) work is underway to develop and validate simpler scoring systems (e.g Asian validation of E-TropICS and Brazilian validation of the SMS-ICU score); that reflect diagnosis and aetiologies more prevalent in such settings, and which attempt to increase score utility, and minimise data missingness.[13,14,15,16]. Despite being of increasing importance in understanding the effectiveness of ICU care on patient recovery, the majority of ICU registries internationally still do not routinely measure long term survival following hospital discharge.[2,18,19]. Despite the increasing presence of electronic health records in high income settings, limited interoperability between in-hospital and social care databases has until recently hindered linking long term outcomes with ICU care. [2,24]. In low and middle income countries (LMICs) practical challenges such as paper records, lack of human resources and communities’ potential distrust of follow up after ICU all hamper efforts to establish follow up services [10] where medium to long term outcomes have been reported survival is less than 50% [24].

### Events of interest

Events of interest typically include complications, or events associated with increased resource use and mortality. A recently published review of registry reported indicators of quality-describes 51 different quality indicators, of which 20 were categorized as outcome events of interest.[7]. The most frequently reported events of interest focused on potentially avoidable adverse events such as healthcare associated infections (HCAIs), pressure sores, duration and complications of invasive mechanical ventilation, prolonged ventilation and unplanned readmission.[4,7,20–23]. Wide variation and inconsistency in collection, definition and reporting of such measures was described, hindering utility and interpretability of such outcomes. Several large scale studies analysing incidence of HCAIs failed to draw meaningful conclusions due to underreporting, missingness of data and inconsistencies in extracting and reporting of data through the registries. [4,10,23,27,28]. These inconsistencies were greatest where extraction was by hand from paper records. Concerns have also been raised as to whether reporting such events actually leads to improvements in care quality. [20]. Events of interest are often used to suggest suboptimal care, however in focusing on the negative, these indicators and their measurement provides little insight for teams seeking to inform actionable improvement or reinforce good practice. Whilst arguably more acceptable to stakeholders, very few of the event based outcomes reported in registries focused on the presence or inclusion of events associated with positive care outcomes.[25–27]. Notable exceptions were the NICE registry in the Netherlands, and EpiMed (Brazil) both of which are using audit and feedback mechanisms inbuilt within electronic registry platforms to try and increase desirable events as part of quality improvement initiatives.

### Resource utilisation and cost

**Figure 1:**
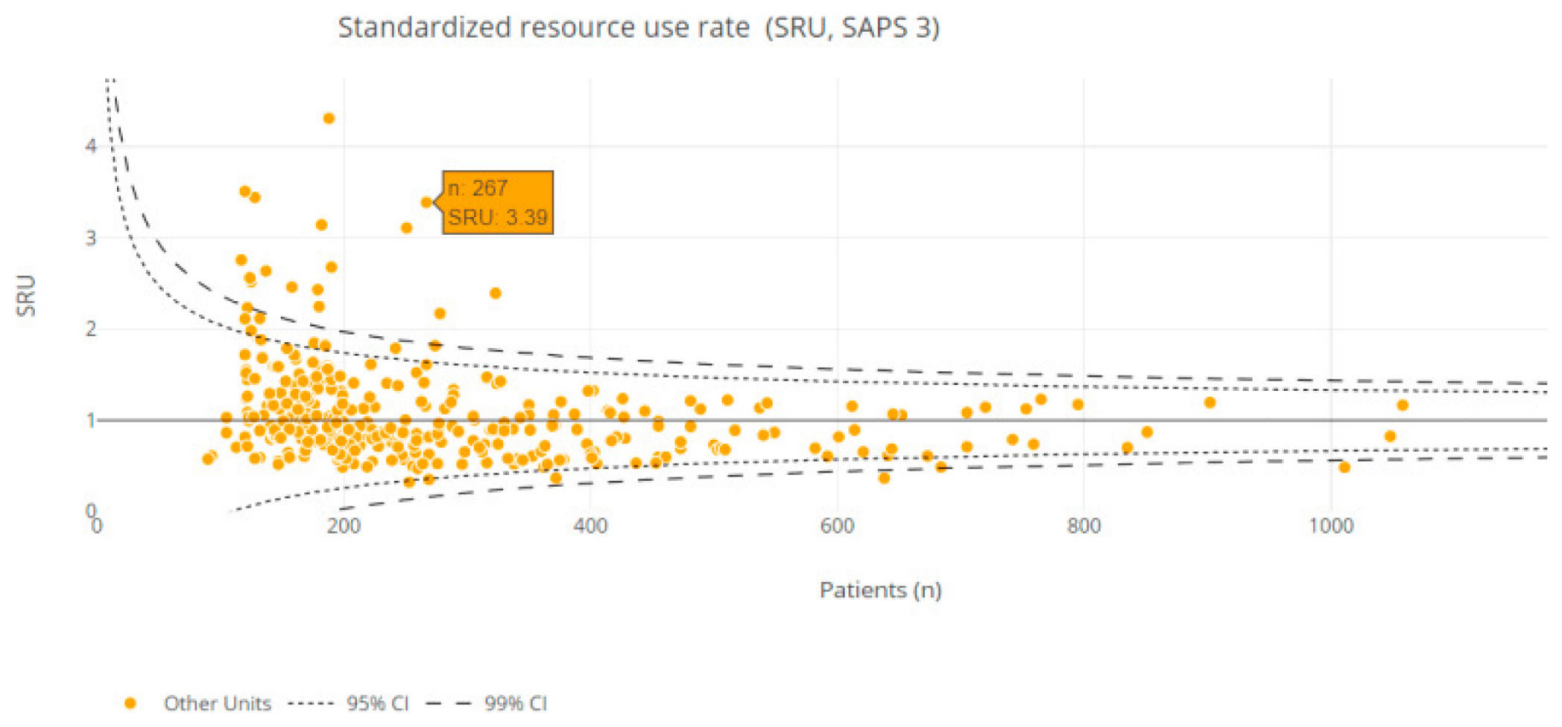
Funnel plot graphic for the benchmarking of ICU resource use. The metric used is the standardized resource use (SRU) based on SAPS 3. Each yellow dot represents an ICU. Lowest rates represent better resource use and efficiency (lower observed/expected resource use rates).

Resource utilization measures capture the patient’s interactions with the healthcare system. Measures including occupancy rates, organ support, medications and ICU and hospital lengths of stay as well as events associated with increased resource utilisation, e.g. readmission to ICU in the same hospital admission are of increasing interest for managers and health systems and have a major impact on patient and family experience of ICU care. [33]. In addition, these measures are used alongside measures of expenditure to determine cost and cost effectiveness of critical care services. [25]. Whilst ICU registries internationally capture the patient centered measures, the potential of organisational structures in evaluating outcomes has only recently begun to be understood. Recent cost effectiveness evaluations from ANZICS have suggested that increased sizes and occupancy of ICU may improve efficiency[29]. The use of standardized metrics may improve the benchmarking on resource use and be considered a proxy of ICU efficiency (Figure 1). For example APACHE IV and SAPS3 scores have been used by registries in the UK, Brazil and Australia to risk-adjusted LOS as a surrogate for resource utilization.[25,40,51]. More recently the COVID-19 pandemic has brought into sharp focus the vulnerability and finite nature of critical care resources globally. Resource and cost data from ICU registries in India was used to provide insights into the impacts of COVID-19 on ICU service utilisation and in doing so highlighted how contextual factors; organisational cultures, team structures may influence resource utilisation and service outcomes. [55] Moving forward ICU registries could provide increasingly valuable contributions to surveillance of ICU resource utilisation, efficiency and the impact of ICU on public health outcomes.

### Patient-Reported Outcome measures

Patient-reported outcome measures (PROMs) reflect the patients’ perceptions of their health status and their perspective on health and disease. [30] PROMs have become an increasingly important avenue for research in ICU given increasing numbers of survivors.[31]. For the most part, PROMs are confined to registry led studies and are not routinely captured in ICU registries. Selection and operationalisation of specific PROMs for use within registries is not, however, straight forward and substantial heterogeneity of capturing and reporting remains. Furthermore, the number of possible PROMs is potentially overwhelming, their definition, character and interpretability complex and their ability to detect change in quality of care over time uncertain. [26]. Burden of data collection on both the patient and for the healthcare team is a major consideration. In LMICs, these concerns are compounded by uncertainty over how patients and healthcare providers currently understand quality of care and how current expectations of care may invalidate such measures.[2] Nonetheless, PROMs are increasingly used, to support quality assurance and improvement initiatives, and may be of particular value in resource constrained settings- to help refine indications for ICU admission, and help close the quality gap.[2]. The use of novel technologies such as mobile application-based questionnaires as well as linkage with electronic medical records may facilitate data collection beyond ICU admission and allow large scale, consistent information on quality of life, return to daily activities and work among other domains (Table 1).

## Challenges in interpreting outcome measures internationally

As previously mentioned, survival remains a central outcome measure in ICUs. Whilst ICU mortality rates and associated measures such as length of stay have steadily decreased across upper and middle income countries over the last 10 years, demand for critical services and ICU admission in these same settings is rising. In contrast ICU mortality in LMICS whilst varying between regions remain disproportionately high (30–80%) and remain inversely correlated to national income levels [4,14,25,27]. In higher income settings, where critical care services are increasingly well established, early referral and admission of patients from wards who require single organ support and admission of patients for optimisation of physiology prior to major surgery has undoubtedly contributed to improved ICU and hospital survival rates. Potentially the increasing availability of transfer from ICU to rehabilitation services for patients following major orthopaedic and neurological injury may influence both ICU survival and ICU lengths of stay. Such investments in acute and chronic care remain largely absent in resource limited settings with significant variation among and within countries. [28,29] As a consequence, ICU in these settings are often used as a terminal destination for patients at the end of life, often following cardiac arrest, or in the presence of long-standing organ failure. [27,30]

To increase complexity further, dying in hospitals in many parts of the world is neither culturally desirable or socially acceptable for patients, their families or for their treating clinicians. In such instances ICU admission may be negotiated by specialists as a way to provide mechanical ventilation as a bridge to family members being able to prepare to take their loved one home to die. [4]. For patients already in ICU, arrangements may be made for transfer from the ICU directly home, perhaps with an endotracheal tube in situ. Indiscriminate reporting of mortality figures in these settings, or worse, attempts to draw comparison internationally is unadvisable. Given the legal, cultural, and socioeconomic factors likely to influence decision-making, and recording and reporting of mortality, greater international multidisciplinary perspective is required. [31]. Conversely, cultural differences may play a role in increasing the use of ICU by terminal patients resulting in both admission of patients with low survival expectations but also on low degree of implementation of palliative and end-of-life care measures that ultimately lead to prolonged, expensive and futile use of intensive care resources.

Crucially clinicians and healthcare users in ICU require support and training to interpret outcomes measures. This may improve trust among clinicians and researchers seeking to improve care using registry data, as well as patients and policy makers. National registries and international initiatives such as the LOGIC (Linking of global intensive care) have an important role in providing standardised metrics and improving the ability for its comparison by using reliable and risk-adjusted measures. [35]

## How intensive care registries can improve implementation and interpretation of ICU outcomes

### Development of standardised outcome measures

Standardised outcome measures provide a set of outcome measures that are feasible to capture in registries that are important to clinical providers, patients and their families. [26]. Standardisation of outcome measures in registries, means variables are mapped to terminologies that facilitate consistent and replicable collection [6]. Support for multiple efforts to develop common data elements for use in registries is growing. The use of established standardized outcome measures is essential so that registries can contribute to evolving evidence and quality improvement practice. Critical care registry projects including “Collaboration for Research, Implementation and Training in Asia and Africa (CCAA)” “Global Open Source Severity of Illness Score”and a study looking at organisational characteristics, outcomes and resource utilisation in Brazil have demonstrated the potential in using large scale registry databases to use data as a driver of quality improvement. [40, 41, 47,48,]. Currently the National Institute of Health, Duke Clinical Research Institute and the Pew Charitable Trust are investing in registry data standards for concepts in registries. Standard terminologies not only improves efficiency when establishing registries but also promotes data sharing, and linking of datasets from different sources[42]; critical for increasing interoperability between registries internationally. In addition, harmonisation of existing critical care data sets using common data models is underway. Pioneering work by groups such as the Observational Health Data Sciences and Informatics (OHDSI) community are working in partnership with ICU registries in the Netherlands, and in Asia to apply a common data models (CDM) to cleanse and standardise existing registry data. [43]. The CDM allows for the increased interoperability of information, systematic analysis of data from different data sources using common representation (terminologies, vocabularies, coding schemes), and then performing systematic analyses using a library of analytical tools. These tools could help enable standardised outcome measures thus improving its ability to be interpreted across diverse populations.

### Building collaboration for benchmarking between registries to promote data sharing

Central to strengthening the quality and utility of ICU outcome measures is improved communication and collaboration between ICU registries and healthcare databases internationally. Perhaps the most comprehensive and current effort towards achieving this is Linking of Global Intensive Care (LOGIC). [46,47,48] A collaboration of registries from 13 countries, it includes aggregate data on over seven million ICU encounters from Asia, Australasia, Europe, North America and South America. Its aim is to promote data sharing and international benchmarking through pragmatic reporting of crude aggregated national data from registries. Whilst efforts to harmonise data sets and standardise outcomes are still underway, LOGIC’s approach to reporting aggregate data in a single common platform is the first step for many ICU registries in collaboration.

### Leveraging mobile and wearable technology to increase the feasibility of measuring medium to long term patient centred outcomes

The increasing implementation of digital registries and their potential to communicate with mobile technology and wearable devices could rapidly accelerate the feasibility of routine and sustainable measurement of PROMs. [49]. Data obtained from questionnaires enabled by mobile applications can and are starting to provide patients and clinicians with near real-time data on medium to long term quality of life, functional recovery and psychological well being.[50]. Centralising information to cloud-based registries is both feasible and resource light. Leveraging already accessible tools could be especially important in middle and lower middle income settings, where absence of long term outcomes is in part perpetuated by the financial and human resources costs associated with long term follow up.

### Actionable outcome measures

Actionable indicators are gaining prominence in the healthcare improvement arena. Data pertaining to ICU outcome measures; specifically events of interest (e.g HCAI rates) are increasingly available through registries dashboards and allow ICUs to compare rates over time. Such measures may have a role in promoting improvement in ICU, providing they are selected, implemented and interpreted in partnership with healthcare providers. The Agency for Healthcare Research and Quality have advocated for outcome measures to be used to improve performance in daily practice. Positive reinforcement from changes in measurement reported over time may complement existing tools for audit and feedback as part of quality improvement initiatives. As described above, both the multi national registry Epimed [51], and Dutch intensive care registry have shown the potential for registries as more than simply reporting ICU outcomes.[52,53]. Both used registry data as part of a feedback loop to reinforce positive behaviours in clinical care as part of quality improvement initiatives. Such methods are likely to become increasingly central to how ICU registries are used in healthcare systems internationally providing the validity, actionability and reproducibility of data from registries can be assured.

## Conclusions

Registries have contributed significantly to the reporting and interpretation of ICU outcome measures internationally. ICU registries have an increasingly central role in benchmarking ICU care, however there are still challenges to implementation and interpretation of current outcome measures. Alongside variation in definitions, implementation and reporting of outcomes in ICU, international comparisons using current measures are making progress. Advances in IT and data science have created opportunities to change the way ICU outcomes are reported and . could additionally support the use of actionable measures to improve patient’s outcomes.

## Figures and Tables

**Table 1:** Current status and future directions regarding outcome measures in ICU registries

Core Outcome Measures		Additional outcome measures	Future Directions
Survival	ICU and Hospital mortality; Standardized mortality rates	30 or 60-day mortality	Long-term survival (6months and 1 year)
Events	Adverse events, Nosocomial infections	Delirium rates, ICU acquired weakness, nonadherence to protocols (EBM measures)	Optimal sedation and analgesia rates;
Resource use	ICULLOS, Use of advanced life support (i.e. MV, RRT)	Hospital LOS, MV free-days, ICU costs,	Severity-adjusted nursing hours,
Morbidity	Co-morbidities,Age	Frailty (i.e.MFI, CFS), Functional capacity	Functional (i.e. Barthel)index at hospital discharge; Post-intensive care syndrome, quality of life (Long-term); Functional (activities of daily living) trajectory; cognitive function

## Key points

Registries provide a pivotal role in reporting ICU outcome measures.

Whilst ICU survival is increasing globally, registries reveal that there is ongoing disparity in outcomes internationally.

Data on long-term mortality and functional outcomes in ICU registries are globally lacking.

Main challenges to interpreting ICU outcomes reported by registries include the evolving role of ICU care in acute and chronic management, limited feasibility of disease severity scores and wide variation in health system infrastructure.

Implementation of medical informatics offers novel possibilities for overcoming current challenges in utilising and interpreting registry reported ICU outcome data
